# MicroRNA-29b/graphene oxide–polyethyleneglycol–polyethylenimine complex incorporated within chitosan hydrogel promotes osteogenesis

**DOI:** 10.3389/fchem.2022.958561

**Published:** 2022-07-22

**Authors:** Han Qin, Yujie Ji, Guangyue Li, Xiaohui Xu, Chuangwei Zhang, Wenjie Zhong, Shihan Xu, Yuanyuan Yin, Jinlin Song

**Affiliations:** ^1^ College of Stomatology, Chongqing Medical University, Chongqing, China; ^2^ Chongqing Key Laboratory of Oral Diseases and Biomedical Sciences, Chongqing, China; ^3^ Chongqing Municipal Key Laboratory of Oral Biomedical Engineering of Higher Education, Chongqing, China

**Keywords:** graphene oxide, gene delivery system, miR-29b, functionalization, osteogenesis, chitosan hydrogel, polyethylenimine, polyethyleneglycol

## Abstract

MicroRNAs (miRNAs) play a pivotal role in regulating a number of physiologic and pathologic processes including bone marrow mesenchymal stem cell (BMSC) osteogenic differentiation, making them a candidate used to promote osteogenesis. However, due to intrinsic structure and characteristics, “naked” miRNAs are unstable in serum and could not pass across the cellular membrane. Nano delivery systems seem to be a solution to these issues. Recently, graphene oxide (GO)-based nanomaterials are considered to be promising for gene delivery due to their unique physiochemical characteristics such as high surface area, biocompatibility, and easy modification. In this work, a GO-based nanocomplex functionalized by polyethyleneglycol (PEG) and polyethylenimine (PEI) was prepared for loading and delivering miR-29b, which participates in multiple steps of bone formation. The nanocomplex revealed good biocompatibility, miRNA loading capacity, and transfection efficiency. The miR-29b/GO-PEG-PEI nanocomplex was capsulated into chitosan (CS) hydrogel for osteogenesis. *In vitro* and *in vivo* evaluation indicated that miR-29b/GO-PEG-PEI@CS composite hydrogel was able to promote BMSC osteogenic differentiation and bone regeneration. All these results indicate that PEG/PEI functionalized GO could serve as a promising candidate for miRNA cellular delivery, and the miR-29b/GO-PEG-PEI@CS hydrogel has the potential for repairing bone defects *in vivo*.

## 1 Introduction

MicroRNAs (miRNAs) are a class of evolutionally conserved, endogenous small non-coding RNAs (approximately 19–25 bases) that could regulate the expression of around 30% of human genes and a number of physiologic and pathologic processes including cell cycle, metabolism, differentiation, and apoptosis (Ambros, 2004) (Braun and Gautel, 2011) (Pillai et al., 2007). By binding to the 3’-untranslated regions of specific targeted mRNAs, miRNAs provoke either translational repression or degradation depending on the degree of sequence homology (Filipowicz, 2005). Currently, knowledge is continuously accumulating related to miRNAs' involvement in the regulation of bone marrow mesenchymal stem cell (BMSC) osteogenic differentiation (Zhang et al., 2021). By conducting miRNA array and profiling/pathway analyses, certain miRNAs have been identified to control the process of BMSC osteogenic differentiation (Zhang et al., 2021) (Fröhlich, 2019). Of those miRNAs, miR-29b has been demonstrated to promote osteoblastogenesis at multiple stages and negatively regulate human osteoclast differentiation and activity (Li et al., 2009) (Rossi et al., 2013). Thus, miR-29b could be a therapeutic target for promoting bone formation in bone defect.

miRNA mimics, which contain exactly the same sequence as the mature endogenous miRNAs and possess the ability to combine with target mRNAs, have been developed as alternative miRNAs to regulate cell activities. However, several issues hinder the application of miRNA mimics for treating diseases (Winkle et al., 2021) (Bai et al., 2019). First, “naked” miRNAs are often considered to be highly unstable due to the renal clearance and rapid degradation by RNase or other molecules in serum (Bai et al., 2019). Second, the intrinsic negative charge of miRNAs interferes with the interaction between miRNAs and the cell membrane, which results in poor cellular uptake (Bai et al., 2019). In order to solve these issues, various strategies have been developed such as a nanodelivery system. The miRNA delivery system should achieve highly efficient cellular uptake and tissue-specific delivery, as well as minimize systemic toxicity (Bai et al., 2019). Until now, a number of viral and non-viral vehicles have been developed for miRNA delivery. Viral vehicles could transfect efficiently into the target cells, but some disadvantages limit their application such as undesired immunogenic/inflammatory responses, the risks of being integrated into the host genome, large-scale manufacturing, low loading capacity, and quality control (Sun et al., 2015; Winkle et al., 2021). Non-viral delivery systems, including liposomes, polymer-based carriers, and inorganic nanoparticles, are designed to overcome those defects (Bai et al., 2019).

Recently, graphene oxide (GO) is considered to be a promising nanomaterial for gene delivery due to its unique physiochemical characteristics such as high surface area, biocompatibility, and easy modification (Vincent et al., 2017) (Mohajeri et al., 2018) (Hoseini-Ghahfarokhi et al., 2020). Studies have used GO-based nanomaterials to load siRNA, miRNA inhibitor, gene, or plasmid for inhibiting tumor growth, vasculogenesis and cardiac repair, blocking osteoclastogenesis, bone regeneration, *etc.* (Yin et al., 2013) (Paul et al., 2014) (Dou et al., 2018) (Ou et al., 2019). In this study, cationic polymer polyethylenimine (PEI) was conjugated to polyethyleneglycol (PEG)-modified GO for miRNA mimic delivery. This miRNA delivery system presents not only good biocompatibility but also considerable loading and delivery efficiency. The osteogenesis-promoting microRNA, miR-29b, was loaded on the nanocarrier GO-PEG-PEI. The miRNA-loaded nanocomplex was successfully transfected into BMSCs. Moreover, this complex was incorporated into a chitosan (CS) hydrogel (miR-29b/GO-PEG-PEI@CS), which was a biocompatible scaffold as we previously reported (Qin et al., 2018). *In vitro* and *in vivo* studies revealed that miR-29b/GO-PEG-PEI@CS could promote osteogenesis and bone regeneration.

## 2 Materials and methods

### 2.1 Materials

PEG functionalized GO (GO-PEG) with a diameter of 190–320 nm was purchased from Nanjing/Jiangsu XFNANO Materials Tech Co., Ltd. (Nanjing, China). Branched PEI with molecular weight (MW) of 25 kDa, N-(3-dimethylaminopropyl-N′-ethylcarbodiimide), and hydrochloride (EDC) were purchased from Sigma-Aldrich (Missouri, United States).

### 2.2 Preparation and characterization of graphene oxide–polyethyleneglycol–polyethylenimine

For the preparation of GO-PEG-PEI, a GO-PEG solution (1 mg ml^−1^) was added with EDC (0.5 mg ml^−1^) and stirred gently at room temperature for 10 min. Then, PEI (1 mg ml^−1^) was mixed into GO-PEG solution, sonicated for 5 min, and stirred at room temperature for 6 h following the second time addition of EDC (0.5 mg ml^−1^). After that, the mixture was washed 3–5 times with deionized water by a 100-nm Milli-Q membrane filter (Millipore, Bedford, MA), obtaining GO-PEG-PEI re-suspended in water.

Elemental analysis data and FTIR spectrum of GO-PEG-PEI were obtained by an elemental analyzer (Vario MACRO cube) and an FTIR spectrometer (Nicolet iS50), respectively. Transmission electron microscopy (TEM, JEM2800F) was used to observe the GO-PEG-PEI nanocomplex. The surface charge and size of GO-PEG-PEI were measured by a Zetasizer instrument (Zetasizer nano zsp). The contents of PEG and PEI were estimated by thermo-gravimetric analysis (TGA, Mettler tga 2).

### 2.3 Cell culture

BMSCs were harvested from the bone marrow of two-week-old Sprague-Dawley (SD) rats that were purchased from the Experimental Animal Center of Chongqing Medical University (Chongqing, China). Cells were cultured in α-minimum essential medium (α-MEM; Hyclone, Logan, UT, United States) supplemented with 10% fetal bovine serum (FBS; Gibco, United States), 100 U/mL of penicillin, and 100 μg/ml of streptomycin.

### 2.4 Cellular toxicity of graphene oxide–polyethyleneglycol–polyethylenimine

The cellular toxicity of GO-PEG-PEI was examined by the CCK-8 assay kit (AbMole, United States). Briefly, BMSCs were cultured on a 96-well plate with a density of 5 × 10^4^ cells/mL. Twenty-four hours after being seeded, different concentrations of GO-PEG-PEI (10, 20, 30, 40, 50, 75, and 100 μg/ml) were added to the cells. After 24 and 48 h, the media was removed, and the mixture of CCK-8 solution and fresh media with a volume ratio of 1:10 was added to each well and incubated for 2 h at 37°C. The absorbance was tested by using a microplate absorbance reader (Bio-Rad, CA, United States) at 450 nm.

### 2.5 Preparation of miRNA/graphene oxide–polyethyleneglycol–polyethylenimine complex and evaluation of microRNA loading capacity

The miR-29b was bought from Tsingke Biotechnology Co., Ltd. (Beijing, China). MiR-29b/GO-PEG-PEI complexes of different N/P ratios (0, 0.5, 1, 2, 5, 10, and 20) were obtained by mixing the appropriate volume of GO-PEG-PEI solution (0.35 mg/ml) and miR-29b solution (10 μM) on ice for 20 min. To determine the microRNA loading capacity of GO-PEG-PEI, the agarose gel electrophoresis assay was conducted. Briefly, the as-prepared miR-29b/GO-PEG-PEI complex solutions (8 μL) were added with 2 μL of 5 × loading buffer and then loaded on a 1% (w/v) agarose gel and electrophoresed at 100 V for 25 min. Then, the gel was imaged by a gel imager (Bio-Rad chemiDoc).

### 2.6 Cellular uptake of miRNA/graphene oxide–polyethyleneglycol–polyethylenimine and transfection efficacy

Cellular uptake of miR-29b/GO-PEG-PEI was observed by laser scanning confocal microscopy (LSCM). Briefly, GO-PEG-PEI was mixed with Cy3-labeled miR-29b (miR-29b-Cy3) solution (Tsingke, Beijing, China) at N/P ratios of 5, 10, 20, 40, and 80. The miR-29b-Cy-3/GO-PEG-PEI complexes were used to incubate with BMSCs for 3 h. Then, cells were observed under a fluorescence confocal microscope (Leica, Germany). The transfection efficacy was measured by quantitative RT-PCR analysis. Various N/P ratios (5, 10, 20, 40, and 80) of miR-29b/GO-PEG-PEI complexes with the same concentration of miR-29b that equaled 10 nM in the final media were incubated with BMSCs for 3 h. Then, total RNA was extracted from BMSCs using RNA Isolation Kit (Beyotime, China). Next, 500 ng of total RNA was used as the template to synthesize the first-strand cDNA of microRNAs using the miRcute Plus miRNA First-Strand cDNA kit (TIANGEN BIOTECH, Beijing, China). For PCR amplification, a 10-μL reaction volume was used, comprising 5 μL of 2 × miRcute Plus miRNA Premix (Cybr&ROX, TIANGEN), 200 nM of forward primer (Tsingke) and reverse primer (TIANGEN), 2 μL of fivefold diluted cDNA, and 2.6 μL of RNase-free water. The reaction and detection were conducted using a CFX96 Real-Time PCR Detection System (Bio-Rad Laboratories, Inc., Hercules, CA, United States). The cycle threshold (Ct) values were collected and normalized to the level of U6, a housekeeping microRNA. The primer sequence used for miR-29b and U6 were as follows: miR-29b (F) 5’-AAC​ACT​GAT​TTC​AAA​TGG​TGC​TA-3’; U6 (F) 5’-ATA​TGG​AAC​GCT​TCA​CGA​ATT-3’.

### 2.7 Preparation of miR-29b/graphene oxide–polyethyleneglycol–polyethylenimine@CS hydrogel

miR-29b/GO-PEG-PEI was encapsulated in a chitosan (CS) hydrogel, which was prepared as the authors reported previously (Qin et al., 2018). Briefly, CS powders were dissolved in acetic acid (0.75%v/v), then miR-29b/GO-PEG-PEI solution was added to the CS solution under stirring, and lastly, a β-glycerophosphate solution was added. After being homogeneously mixed, the final solution was tiled on a Teflon plate and gelled at 37°C.

### 2.8 ALP activity

To induce the BMSC differentiation, cells were seeded at a density of 1 × 10^5^ cells per well in a 6-well culture plate and incubated with miR-29b/GO-PEG-PEI on CS hydrogel and CS hydrogel encapsulating miR-29b/GO-PEG-PEI (miR-29b/GO-PEG-PEI@CS). When the cells reached 80% confluency, the culture media was replaced with stem osteogenic differentiation medium [α-MEM supplemented with 10% FBS, 1% antibiotics, 50 μM ascorbic acid (Sigma, Missouri, United States), 10 mM β-glycerol phosphate (Sigma), and 0.1 μM dexamethasone (Sigma)]. After 1, 4, and 7 days of osteogenic incubation, alkaline phosphatase (ALP) activity was used to evaluate the osteogenic differentiation of BMSCs by using an ALP analysis kit (Beyotime, China) according to the manufacturer’s instructions. The OD value at 405 nm indicated the ALP activity.

### 2.9 Animals

Animal experiments in this study were conducted in accordance with the Declaration of Helsinki, ARRIVE guidelines, and the National Institutes of Health Guide for the Care and Use of Laboratory Animals under protocols reviewed and approved by the Ethics Committee of the College of Stomatology, Chongqing Medical University. Sprague-Dawley (SD) rats were purchased from the Experimental Animal Center of Chongqing Medical University (Chongqing, China). All animals were housed in a specific pathogen-free room at the Chongqing Key Laboratory of Oral Diseases and Biomedical Sciences and received water and food *ad libitum* from the Animal Care Facility Service.

Six-week-old SD rats (*n* = 5) were used to create a skull defect model of 5 mm in diameter. The as-prepared miR-29b/GO-PEG-PEI@CS and CS hydrogel were placed to cover the defect before suturing the wound. Two months later, the rats were sacrificed, and the top part of the skull with the defect model was harvested.

### 2.10 Microcomputed tomography

The harvested cranial specimen was scanned by using a high-resolution CT system (Skyscan 1,172; Skyscan, Aartselaar, Belgium). Scans were performed using the following scanner settings: X-ray source voltage 70 kVp, current 114 μA, and power 8 W. CT scan (Scanco Medical AG) settings were high resolution, voxel size of 15 μm, slice thickness of 0.01 mm, and FOV/diameter of 30.7 mm, while an integration time of 250 ms micro-CT was used to assess the regeneration of cranial bone. To quantify bone regeneration, the projection of the bone defect model on the Z-axis was achieved by using Fuji software. Then, a circle of 5 mm in diameter around the defect was chosen as the interested zone. By measuring the grey area which indicates bone in the circle, the ratio of new bone to defect can be calculated.

### 2.11 Histological analysis

The obtained samples were fixed with 4% paraformaldehyde overnight, decalcified in 10% ethylenediaminetetraacetic acid (EDTA) (pH = 8.0) at 37°C for 1 month, and changed medium every 2 days. Dehydrate with a series of ethanol and embed in paraffin. Then, 5-μm-thick paraffin sections were prepared according to the instructions for H&E staining to observe cranial bone regeneration. Digital images were captured under polarized light microscopy (BX41, Olympus, Tokyo, Japan).

### 2.12 Statistics

All experiments were repeated at least three times to confirm the reliability of the study. Data were submitted as mean ± standard deviation (SD). Statistical significance was analyzed by one-way ANOVA among groups or by Student’s t-test between two groups using SPSS (version 24.0; SPSS, Chicago, IL). It was considered statistically significant when *p* < 0.05.

## 3 Results

### 3.1 Characterization of graphene oxide–polyethyleneglycol–polyethylenimine

**FIGURE 1:**
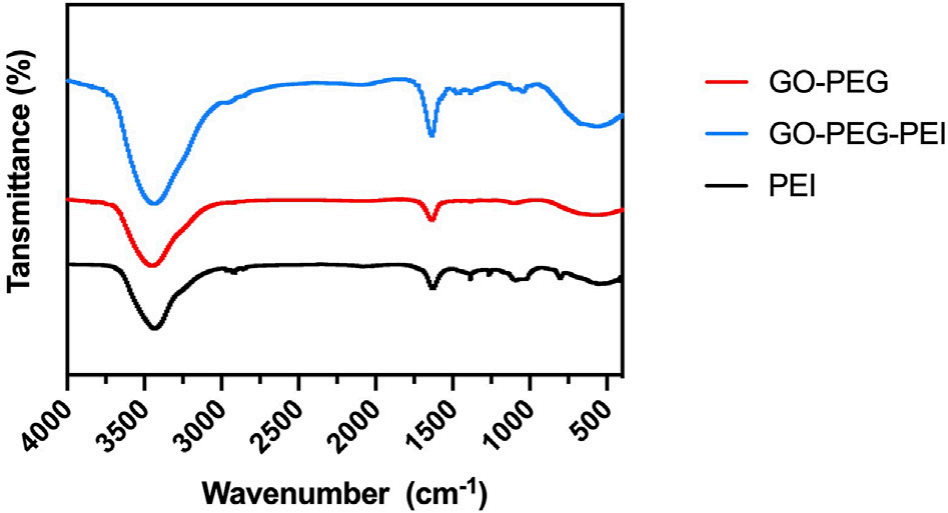
FTIR spectra of GO-PEG (red), PEI (black), and GO-PEG-PEI (blue).

**FIGURE 2:**
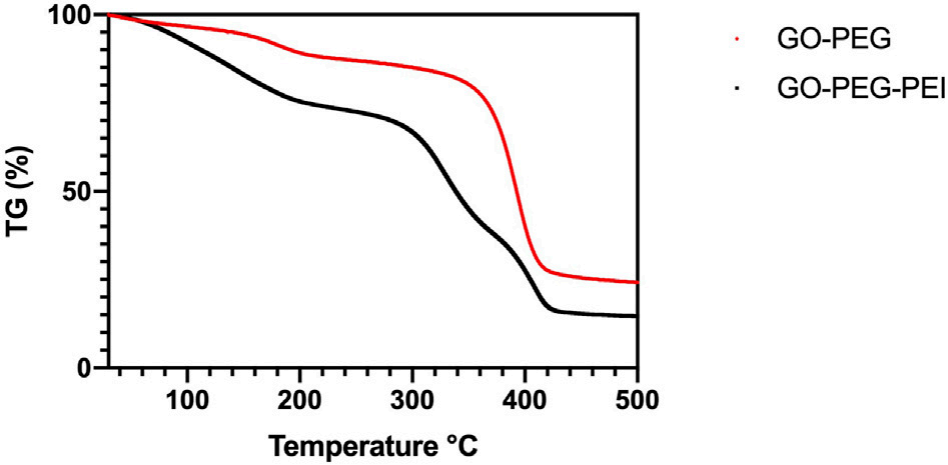
TGA of GO-PEG (red) and GO-PEG-PEI (black).

**FIGURE 3:**
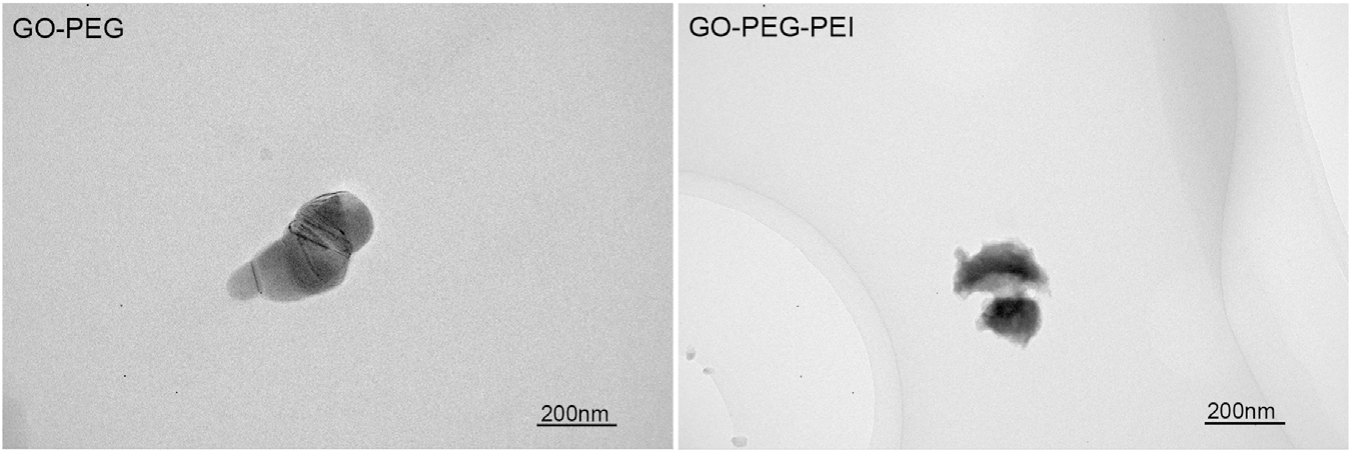
TEM images of GO-PEG and GO-PEG-PEI complexes. Scale bars: 200 nm.

The successful synthesis of GO-PEG-PEI was evidenced by a series of characterizations. As shown in Figure 1, the result of FTIR confirms the covalent binding of PEI and GO-PEG. The peaks at 3,400 cm^−1^ and 1,643 cm^−1^ were related to the O-H and C=O, respectively. The peaks at 2,953 cm^−1^ and 2,875 cm^−1^ were attributed to the CH_2_ vibration of PEI, which could be observed in the spectrums of GO-PEG-PEI and PEI. The reinforced peak at 1,643 cm^−1^ and the peak at 1,410 cm^−1^ were attributed to C=O and C-N of the amide group in the spectrum of GO-PEG-PEI. The characteristic peaks of the amide group and CH_2_ bond in the spectrum of GO-PEG-PEI indicated that PEI was covalently bound to GO-PEG successfully. Dynamic light scattering (DLS) analysis showed the average sizes of GO-PEI and GO-PEG-PEI were 219.06 ± 1.24 and 274.3 ± 2 nm, respectively (Table 1). The zeta potential of GO-PEG and GO-PEG-PEI were -26.9 ± 0.85 and 43 ± 1.33 mV, respectively (Table 1). Both increased size and positive charge of GO-PEG-PEI could be attributed to the binding of PEI. Elemental analysis revealed the nitrogen content of the GO-PEG-PEI complex was 13.51%, which indicated that PEI content in NGO-PEG-PEI was about 41.49% (Table 2). The PEG content of GO-PEG-PEI was estimated to be about 22.28% by the thermo-gravimetric analysis (TGA) (Figure 2). TEM revealed that GO-PEG were flakes with clear and sharp contours, while the outline of GO-PEG-PEI seemed coarser, which could also be attributed to the successful conjugation of branched PEI (Figure 3).

**TABLE 1:** Zeta potentials and average sizes of GO-PEG and GO-PEG-PEI.

Group	Zeta potential (mV)	Size (nm)
GO-PEG	−26.9 ± 0.85	219.06 ± 1.24
GO-PEG-PEI	43 ± 1.33	274.3 ± 2

**TABLE 2:** Elemental analysis of GO-PEG-PEI.

Group	C (%)	H (%)	N (%)	O (%)
GO-PEG-PEI	44.06	9.84	13.51	32.59

### 3.2 *In vitro* biocompatibility of graphene oxide–polyethyleneglycol–polyethylenimine

**FIGURE 4:**
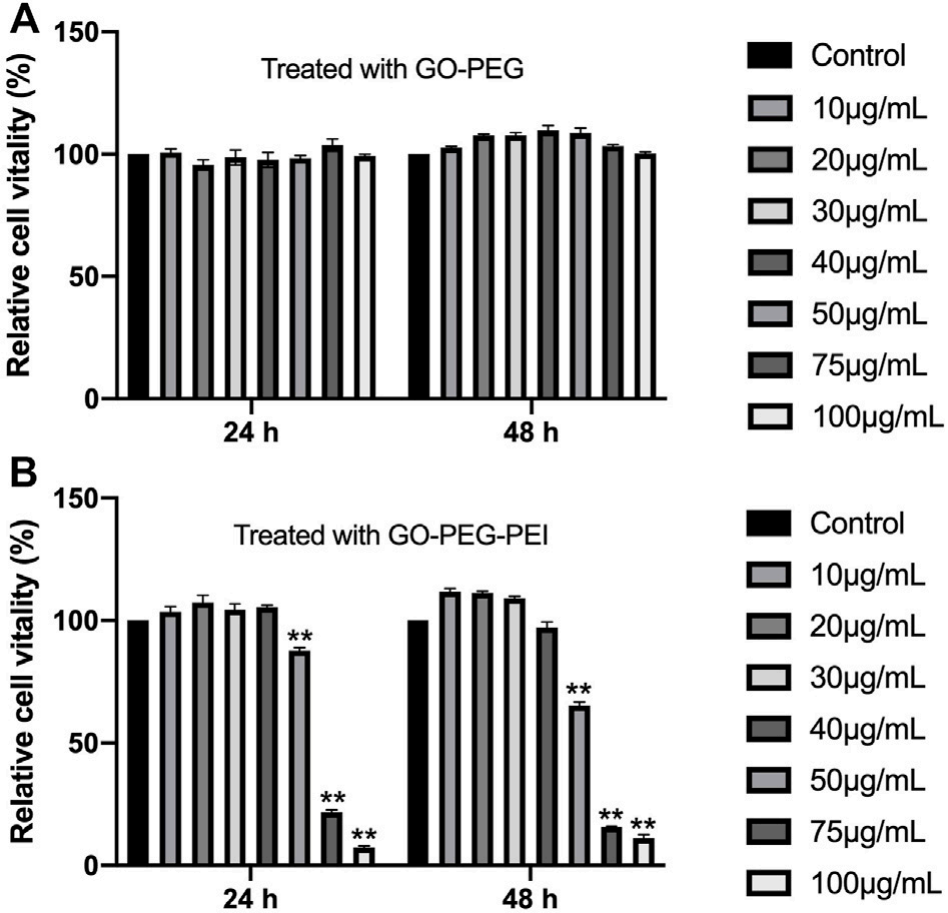
Viability of BMSCs measured by the CCK-8 assay after incubation with various concentrations of GO-PEG **(A)** and GO-PEG-PEI **(B)** for 24 and 48 h. **p < 0.01.

To test the cytotoxicity of the GO-PEG-PEI complex, a CCK-8 assay was performed for BMSCs cocultured with GO-PEG-PEI at different concentrations. GO-PEG revealed good biocompatibility even at a high concentration (100 μg/ml) as indicated in Figure 4A. With conjugation of PEI, the complex mildly inhibited cell proliferation at relatively higher concentrations (50 μg/ml), while showing no obvious toxicity at lower concentrations (≤40 μg/ml) (Figure 4B).

### 3.3 MicroRNA loading of graphene oxide–polyethyleneglycol–polyethylenimine

**FIGURE 5:**
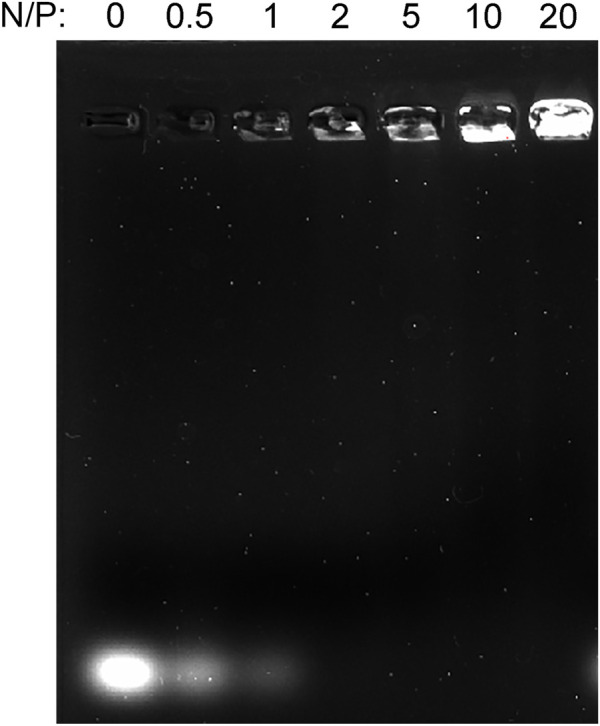
Gel retardation assay of the mixture of GO-PEG-PEI and miRNA at different N/P ratios (0, 0.5, 1, 2, 5, 10, and 20).

Agarose gel electrophoresis was used to assay the affinity or binding capacity for miRNA. GO-PEG-PEI was complexed with miRNAs at various N/P ratios (molar ratio of nitrogen of PEI to phosphate groups of miRNA). As Figure 5 indicates, the miR-29b/GO-PEG-PEI complex demonstrated obvious electrophoretic retardation at the N/P ratio from 2 to 20. This meant that GO-PEG-PEI completely absorbed miRNAs and would prevent them from being degraded when reaching a certain concentration.

### 3.4 Cellular uptake and transfection efficiency of GO-PEG-PEI/miR-29b

**FIGURE 6:**
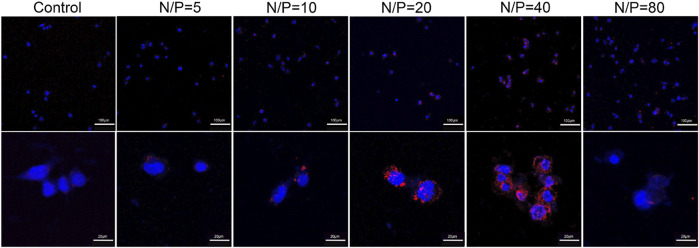
Fluorescent images of miR-29b-Cy3/GO-PEG-PEI complex with different N/P ratios (0, 5, 10, 20, 40, and 80) after 3 h of incubation. miR-29b was labeled using Cy3 (orange-red), and the cell nuclei were stained with DAPI (blue). Scale bars of upper images were 100 μm, scale bars of lower images were 20 μm.

**FIGURE 7:**
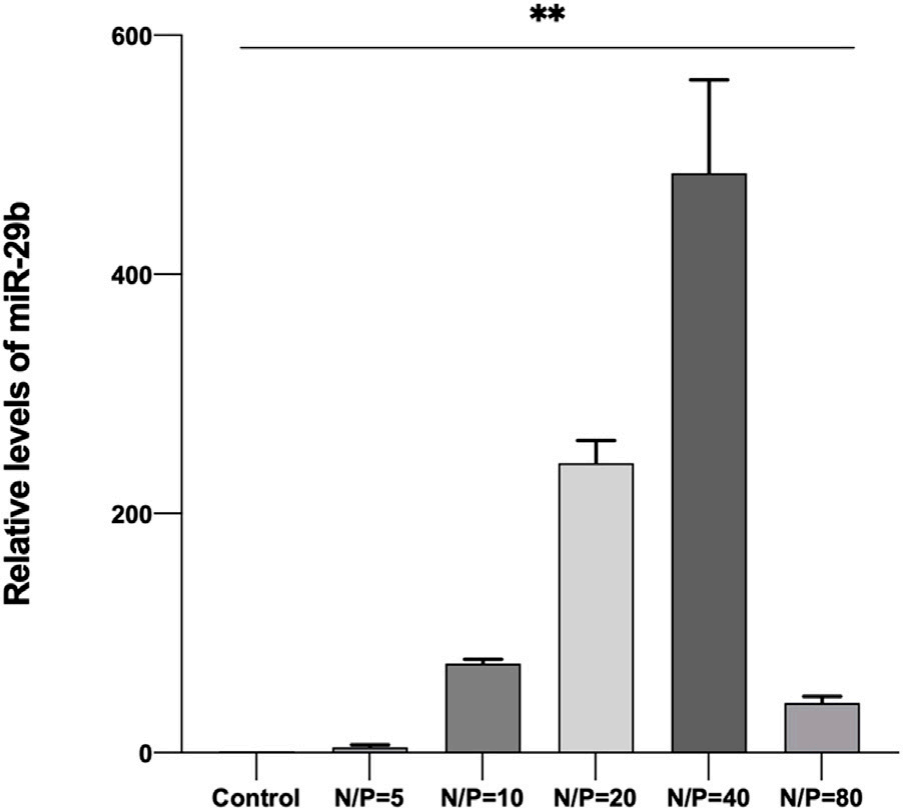
Relative quantity of miR-29b in BMSCs after co-incubation with miR-29b/GO-PEG-PEI complex with different N/P ratios (0, 5, 10, 20, 40, and 80) for 3 h. **p < 0.01.

By using LSCM, miR-29b-Cy3/GO-PEG-PEI was detected in the cell cytoplasm, and some were located within the nucleus (Figure 6). Moreover, miR-29b-Cy3/GO-PEG-PEI with different N/P ratios revealed different transfection efficiencies. As seen in Figure 6, more Cy3-labeled microRNAs were observed in the cells treated with miR-29b-Cy3/GO-PEG-PEI at N/P ratios of 20 and 40. To quantitatively measure the transfection efficiency, quantitative RT-PCR was conducted. The results were consistent with the observation of LSCM. The group of N/P ratio of 40 showed the highest level of miR-29b, followed by the group of N/P ratio of 20 (Figure 7). These results indicated that miRNA/GO-PEG-PEI could be transfected efficiently in a proper N/P ratio. The miR-29b/GO-PEG-PEI complex with an N/P ratio of 40 was used for further experiments.

### 3.5 ALP activity

**FIGURE 8:**
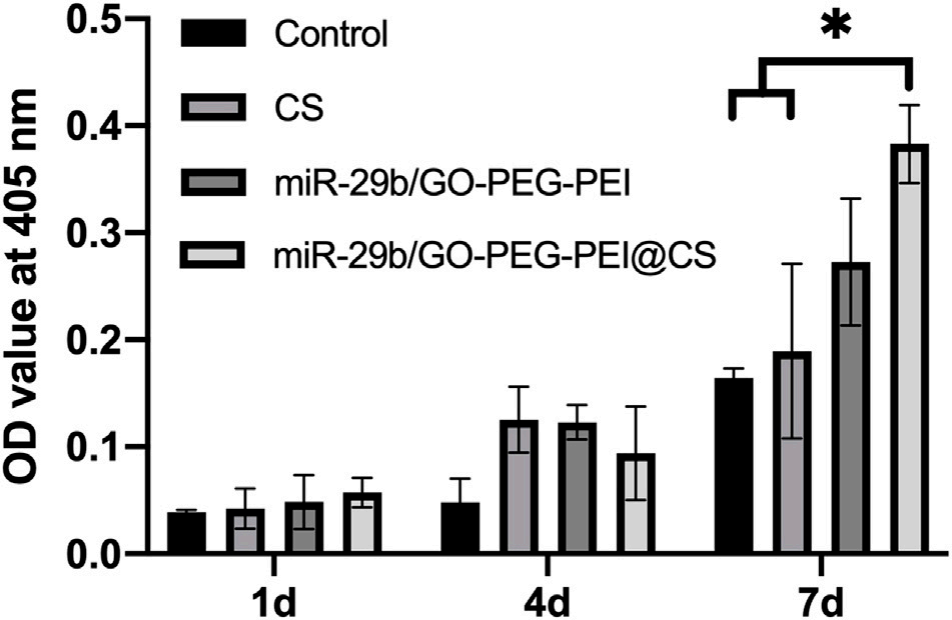
ALP activity of BMSCs. *p < 0.05.

The ALP activity test indicated the osteogenic differentiating level of BMSCs treated with miR-29b/GO-PEG-PEI, CS, and miR-29b/GO-PEG-PEI@CS remained similar at 1 d and 4 d. At 7 d, BMSCs treated with miR-29b/GO-PEG-PEI showed significantly higher ALP activity than CS and control (Figure 8). The ALP activity of BMSCs treated with miR-29b/GO-PEG-PEI seemed to be higher than groups of control and CS, but the difference was significant.

### 3.6 *In vivo* osteogenesis

**FIGURE 9:**
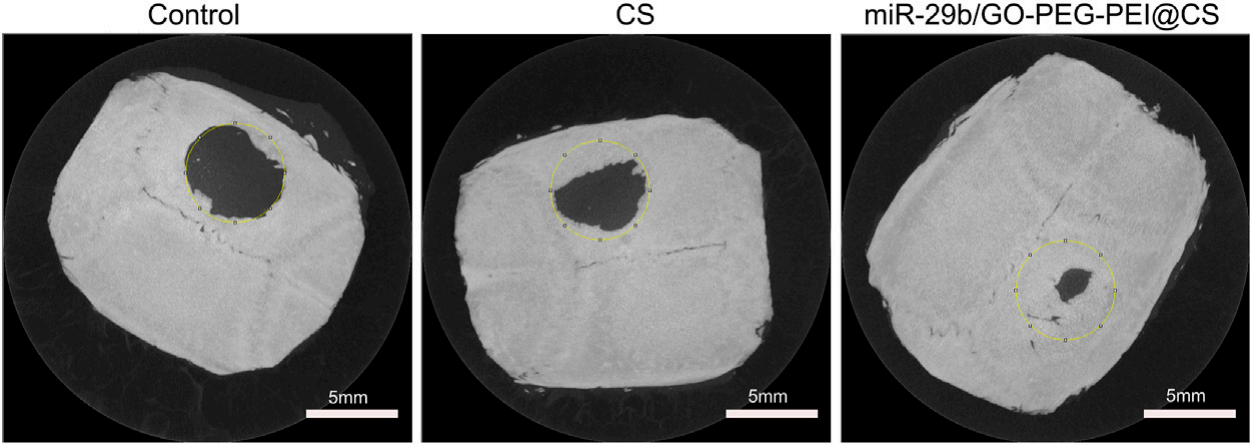
Typical microCT images of rats’ skull defect without material covering or being covered by CS or miR-29b/GO-PEG-PEI@CS hydrogels after 2 months.

As seen in Figure 9, the circles with yellow line indicate the original bone defect of 5 mm in diameter. Apparently, the gray area in the circle of miR-29b/GO-PEG-PEI@CS was the biggest among the three groups, indicating more new bones were formed in the group of miR-29b/GO-PEG-PEI than in the groups of CS and control. By calculating the proportion of gray area in the circle to the total area of the circle, 67 ± 3.5% of the bone defect was covered by new bone in the group of miR-29b/GO-PEG-PEI@CS, while 38 ± 4% of the bone defect was filled with new bone in the group of CS. In the control, merely 10 ± 1.5% of new bone was formed in the defect zone. By histological section and H&E staining, newly formed tissues could be observed above the defect (Figure 11). Apparently, new bone can be observed in the defect model zone of CS and miR-29b/GO-PEG-PEI@CS groups, while the control group presented merely a thin soft tissue above the defect (Figure 11). Moreover, more regenerated bone seemed to fill the defect zone of group miR-29b/GO-PEG-PEI@CS presented than in group CS, as the distance of the defect edges was shorter in group miR-29b/GO-PEG-PEI@CS. These results indicated that miR-29b/GO-PEG-PEI@CS possessed good potential to promote bone regeneration.

**FIGURE 10:**
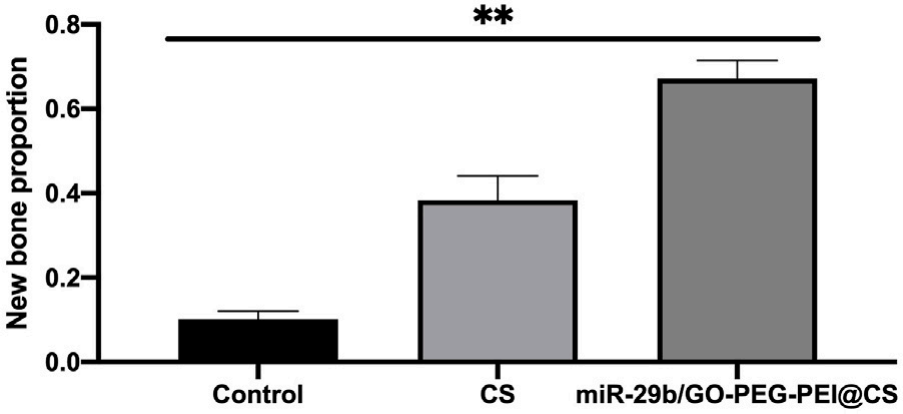
Quantitative assessment of bone regeneration in rats’ skull defect. **p < 0.01.

**FIGURE 11:**
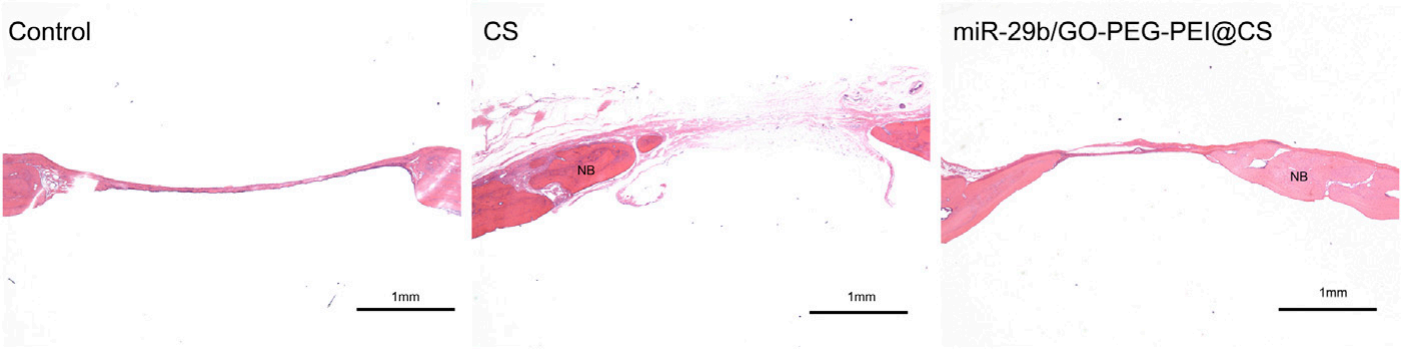
Typical histological sections of rats skull defects in control, CS and miR-29b/GO-PEG-PEI@CS groups. Samples were stained with H&E. NB indicates new bone. Scale bar is 1 mm

## 4 Discussion

Since microRNAs play a pivotal role in tuning biological processes such as immune cell development and function, immune disorders, neural development, and neurological diseases, microRNA-targeted therapies represent a very promising approach for the treatment of various diseases (Winkle et al., 2021). Moreover, it is now well established that miRNAs are physiologically relevant to all steps of bone formation including embryonic development and maintenance during adulthood (Fröhlich, 2019). Of all the microRNAs relevant to bone biology, miR-29b was found to promote osteoblastogenesis at multiple stages as a key regulator due to its versatile effects (Fröhlich, 2019). For instance, miR-29b could silence the negative regulators of osteogenic differentiation such as TGF-β3, HDAC4, ACTVR2A, CTNNBIP1, and DUSP2 (Li et al., 2009). Those negative regulators inhibit signaling pathways such as RUNX2, SMAD, ERK, p38 MAPK, and WNT, which are associated with osteogenic function (Li et al., 2009). In addition, miR-29b could also suppress the synthesis of extracellular matrix proteins such as COL1A1, COL5A3, and COL4A2 that are relevant to bone development, preserving mature osteoblasts in the differentiated phenotype during mineralization, which would enhance mineral deposition and prevent fibrosis (Fröhlich, 2019). Furthermore, some studies also disclosed a link between miR-29b and osteoclastogenesis, indicating that miR-29b is a negative regulator of human osteoclast differentiation and activity (Rossi et al., 2013). Thus, the versatile effects of miR-29b make it a possible target for bone regeneration.

However, due to the intrinsic structure and negative charge, “naked” miRNAs are unable to pass across cellular membrane spontaneously and might be rapidly degraded by RNase in the serum. A nanocarrier system for miRNA delivery is considered a potential approach to solve this issue. In recent years, graphene and its various derivatives have been developed as drug, protein, and nucleic acid delivery systems due to their advantages such as flexibility in design, high mechanical strength, low cytotoxicity, high surface area, easy functionalization with targeting ligand, and high intrinsic mobility (Hoseini-Ghahfarokhi et al., 2020). However, there are still challenges to the application of graphene-based delivery systems. The first one is the possible side effect or cytotoxicity of the nanocarriers. Various factors could affect the cytotoxicity of graphene-based materials such as lateral dimensions, surface area, surface chemistry, surface charge, layer number, purity, particulate state, and shape (Qin et al., 2018). By chemical modification such as conjugating PEG, the biocompatibility of the nanocomplex could be improved (Chai et al., 2019). In this study, the functionalization of GO by PEG revealed good cytocompatibility, and the conjugation of PEI just presented mild cytotoxicity at a concentration of 50 μg/ml (Figure 4B). Second, although graphene or graphene oxide could bind RNA or DNA through hydrophobic and π-π stacking interaction between the ring structures in the nucleobases and the hexagonal cells of graphene (Tang et al., 2015), those interactions are quite weak and unstable, especially in water solution. Moreover, the oxygen-containing groups endow graphene oxide with a negative charge on the surface, which would obstruct the interaction with the negatively charged cellular membrane. In this study, the PEG/PEI dual-functionalized GO was positively charged (Table 1), which potentiated it to absorb negatively charged miRNA by electrostatic force and to protect miRNA from being degraded by RNase in the serum (Figure 5). In addition, the positively charged property and proper size (∼300 μm) could also facilitate the nanocomplex to fuse with the cellular membrane and be digested by the cell (Table 1; Figure 6) (Varma et al., 2022).

Although GO-based nanocarriers present considerable delivery efficiency, controversy still exists regarding their application. The biggest debate is whether graphene or other carbon nanomaterials could not be degraded biologically. Several studies have reported the enzymatic degradation of graphene and its derivatives. Kurapati et al. demonstrated that GO could be degraded by myeloperoxidase in the presence of H_2_O_2_ or by recombinant eosinophil peroxidase (EPO) enzyme extracted from human eosinophils in the presence of a low concentration of hydrogen peroxide and NaBr (Kurapati et al., 2015) (Kurapati et al., 2021). They also found that pristine graphene can be degraded either by recombinant human myeloperoxidase (hMPO) or by hMPO secreted by activated neutrophils (Kurapati et al., 2018). Kotchey et al. (2011) found that GO could be degraded by low concentrations of horseradish peroxidase leading to the appearance of holes on its surface. Mukherjee et al. indicated GO sheets of differing lateral dimensions could be effectively degraded by neutrophils through an MPO-dependent mechanism (Mukherjee et al., 2018). Also, the degradation products were found to be non-cytotoxic and did not elicit any DNA damage (Mukherjee et al., 2018). These results indicate that our immune system has strategies to degrade graphene and GO materials. However, as GO is often functionalized to mitigate the toxicity for biomedical applications, the functionalization may also make the nanomaterials difficult to biodegrade (Chen et al., 2017). For instance, GO modified by bovine serum albumin or PEG could reduce the cytotoxicity but inhibited the activity of horseradish peroxidase (Li et al., 2014). Because these molecules on the GO surface might interfere with interactions between the GO sheet and the enzyme by spatial hindrance (Li et al., 2014). The functionalization and biodegradability seem to be a paradox. So far, most studies concerning GO biodegradability were carried out in microbes or *in vitro*, and the number of microbes and enzymes that have been found to be involved in nanomaterial biodegradation remains limited. Further relevant studies especially long-term *in vivo* studies are needed.

Another question that needs further study is the mechanisms of how GO-based nanomaterials enter cells, which is still not fully understood. Several studies have demonstrated the pathways through which GO enters cells. Yue et al. showed that GO could be phagocytosed by macrophages mediated through IgG-FcgR interaction (Yue et al., 2012). Linares et al. revealed that GO was internalized by osteoblasts and hepatocytes through micropinocytosis or pathways dependent on microtubules and clathrin (Linares et al., 2014). Alnasser et al. showed that the internalization of graphene materials by the cells was mediated by the interaction of key protein recognition motifs presented on the surface of nanomaterials such as apolipoprotein A-I and specific cell receptors like scavenger receptors B1 (Alnasser et al., 2019). Kucki et al. showed that the internalization of GO is highly dependent on the cell differentiation status of the human intestinal cell line Caco-2 due to the different topography of cells (Kucki et al., 2017). All these studies stressed the properties of graphene materials, such as mechanical strength, the amounts of oxygen-containing groups, lateral dimension, thickness, etc., have a non-negligible effect on the pathways of cellular internalization. The functionalization of PEG and PEI changed the surface physiochemical characteristics such as electrical properties, which would have an influence on pathways how GO-PEG-PEI enters cells. Thus, this issue requires further mechanistic studies.

In order to promote osteogenesis *in vitro* and bone regeneration *in vivo*, the prepared miR-29b/GO-PEG-PEI complexes were encapsulated within a chitosan hydrogel. Chitosan has been proved to be a biocompatible and degradable natural polymer for a broad range of applications such as wound dressing, tissue engineering scaffold, drug delivery system, etc. (Jayakumar et al., 2011; Chen et al., 2013; Yang et al., 2014). In the authors’ previous work, chitosan/graphene oxide composite hydrogel displayed proper physiochemical and biological properties, which met the requirements as a scaffold or drug delivery system for repairing bone defects (Qin et al., 2018). In this work, by using a similar method, CS hydrogel containing PEG and PEI functionalized GO nanocomplex loaded with miR-29b were synthesized. The addition of miR-29b/GO-PEG-PEI complexes endowed the hydrogel with the ability to promote osteogenic differentiation and bone regeneration *in vivo* (Figures 8–11). A shortcoming of the material was that the degrading rate was slower than the regenerating rate of new bone. As shown in Figure 11, little material could be found above the bone defect and the new bone did not completely fill the defect after 2 months (Figure 9, Figure 11). This is a challenge for designing an ideal scaffold for repairing bone defects. Nature-derived polymer materials such as collagen and chitosan possess good biocompatibility and biodegradability but are also not strong enough and are degraded too fast (Bharadwaz and Jayasuriya, 2020). Synthetic polymers such as poly (lactic-co-glycolic) acid (PLGA) and polycaprolactone (PCL) are easy to tune their properties like strength but lack bioactivity and are usually degraded slowly (Bharadwaz and Jayasuriya, 2020). Functionalization or composite seems to be the solution to this puzzle (Rossi and van Griensven, 2014) (Jahan and Tabrizian, 2016). In our works, chitosan was composed with GO-based materials with improved physiochemical properties and bioactivities. Encouragingly, the composite hydrogel promoted bone regeneration without causing obvious inflammation, which might be attributed to the functionalization of GO. GO was reported to cause inflammatory responses while some studies indicated PEG functionalized GO could reduce the production of reactive oxygen species (ROS) and stimulate the secretion of the anti-inflammatory cytokine IL-10 (Yue et al., 2012) (Khramtsov et al., 2021).

## 5 Conclusion

In summary, PEG/PEI dual-functionalized GO nanocomplex with biocompatibility was prepared to load miR-29b, which participates in multiple aspects of bone formation. The characteristics of the nanocomplex were revealed by FTIR, TEM, TGA, and zeta potential analysis. The nanocomplex showed acceptable biocompatibility and successfully transfected miR-29b into BMSCs. Chitosan hydrogel was prepared to encapsulate the nanocomplex loading miR-29b. *In vitro* and *in vivo* studies indicated that the composite hydrogel was able to promote BMSC osteogenic differentiation and bone regeneration without causing inflammatory responses. Taken together, the results of this study showed that the miR-29b/GO-PEG-PEI@CS hydrogel is a potential candidate for repairing bone defects *in vivo*.

## Data Availability

The original contributions presented in the study are included in the article/Supplementary Material; further inquiries can be directed to the corresponding author.
